# Phosphatidylinositol-3-phosphate is light-regulated and essential for survival in retinal rods

**DOI:** 10.1038/srep26978

**Published:** 2016-06-01

**Authors:** Feng He, Melina A. Agosto, Ivan A. Anastassov, Dennis Y. Tse, Samuel M. Wu, Theodore G. Wensel

**Affiliations:** 1 Verna and Marrs McLean Department of Biochemistry and Molecular Biology , Baylor College of Medicine, One Baylor Plaza, Houston, TX 77030, USA; 2 Department of Ophthalmology, Baylor College of Medicine, One Baylor Plaza, Houston, TX, 77030, USA; 3 School of Optometry, The Hong Kong Polytechnic University, Hong Kong SAR, China

## Abstract

Phosphoinositides play important roles in numerous intracellular membrane pathways. Little is known about the regulation or function of these lipids in rod photoreceptor cells, which have highly active membrane dynamics. Using new assays with femtomole sensitivity, we determined that whereas levels of phosphatidylinositol-3,4-bisphosphate and phosphatidylinositol-3,4,5-trisphosphate were below detection limits, phosphatidylinositol-3-phosphate (PI(3)P) levels in rod inner/outer segments increased more than 30-fold after light exposure. This increase was blocked in a rod-specific knockout of the PI-3 kinase Vps34, resulting in failure of endosomal and autophagy-related membranes to fuse with lysosomes, and accumulation of abnormal membrane structures. At early ages, rods displayed normal morphology, rhodopsin trafficking, and light responses, but underwent progressive neurodegeneration with eventual loss of both rods and cones by twelve weeks. The degeneration is considerably faster than in rod knockouts of autophagy genes, indicating defects in endosome recycling or other PI(3)P-dependent membrane trafficking pathways are also essential for rod survival.

The roles of phosphoinositides in the function of vertebrate photoreceptors have been studied for decades without the emergence of a clear picture. In recent years, attention has focused on the triply phosphorylated phosphoinositide, phosphatidylinositol-3,4,5-trisphosphate (PIP_3_)^1^,^2^; however, deletion of p85, an essential subunit of the phosphatidylinositol 3-kinase required to synthesize this lipid from the much more abundant phosphatidylinositol-4,5-bisphosphate (PI(4,5)P_2_), has no apparent effect on rods^1^. In addition, it has been proposed that phosphatidylinositol-3-phosphate (PI(3)P) plays a critical role in rhodopsin trafficking to rod outer segments^3^. Phosphatidylinositol-3,4-bisphosphate (PI(3,4)P_2_), another product of Class I PI-3 kinase, has also been proposed to play important roles in cell regulation^4^,^5^, but has not been studied in rods.

Membrane trafficking plays a critical role in rod cell function^6^,^7^, and phosphoinositides are known to play a critical role in formation, sorting, and processing of membranes^8^. In particular, autophagy, a process vital for maintaining cell viability in the face of nutrional stress or deterioration of organelle quality, is known to be essential for rod cell survival^9^,^10^ and to depend on PI(3)P^11^,^12^,^13^. PI(3)P also plays a critical role in endosome processing^14^,^15^, but the roles of endocytosis and endosome processing in rods have not been extensively studied. Previously there have been no measurements of the levels of 3-phosphorylated inositides in rods, and no information was available on the dynamics of these lipids and whether they are affected by light. The studies described here were designed to fill these gaps in understanding the functions and regulation of PI(3)P, PI(3,4)P_2_, and PIP_3_ in retinal rod cells.

## Results

### PI(3)P levels in rod inner/outer segments increase dramatically in response to light

**Figure 1:**
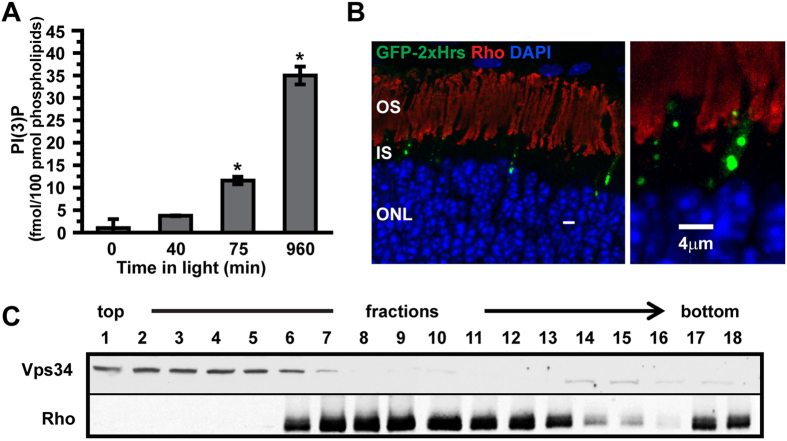
Vps34 and PI(3)P in rods. (**A**) A modified ELISA assay was used to quantify PI(3)P in isolated rod inner/outer segment fragments. The level of PI(3)P increased greatly but slowly during exposure to room light. Data are mean ± SEM of three experiments, each performed in triplicate. * indicates a significant difference compared to all other time points (p < 0.001). For details, see *Materials and methods*. (**B**) PI(3)P was localized to puncta in the inner segment, as visualized by fluorescence of GFP-2xHrs introduced via plasmid. Staining with antibodies to outer segment marker rhodopsin (Rho) reveals PI(3)P is excluded from the outer segment. (**C**) Optiprep gradient fractionation of isolated rod fragments. Each fraction was analyzed by SDS PAGE and immunoblotting for the indicated antigens.

To determine whether 3-phosphorylated phosphoinositides are dynamically regulated by light, we developed a highly sensitive assay based on phosphoinositide binding domains and ELISA with chemiluminescence detection that is capable of quantifying low-abundance phosphoinositides at the femtomolar level (see Materials and methods for details). The results (Fig. 1) revealed a very dramatic (>30-fold) increase of PI(3)P in rod inner/outer segments isolated from light-adapted animals, compared to dark-adapted animals. In contrast, levels of PI(3,4)P_2_ and PI(3,4,5)P_3_ were below reliable detection limits (<0.001%) in either light or dark, with no light-driven increases observed in our experimental conditions (Supplementary Fig. S1).

We measured PI(3)P levels as a function of time during light exposure, to determine whether the light-induced surge in PI(3)P levels is a rapid response to light onset involved in light adaptation of the phototransduction cascade or a longer term stress response to prolonged light exposure. A time course of the PI(3)P increase (Fig. 1A) reveals that the levels increase slowly over a time course of many hours. Thus, these changes likely do not play an important role in the characterized types of adaptation of photoresponses, which occur much more rapidly^16^, implying that the PI(3)P surge is involved in regulation of cellular homeostasis in response to continuous light.

### PI(3)P in rod cells is localized primarily to inner segment puncta

We next asked where the synthesized PI(3)P pools are located. The biochemical analyses were carried out on isolated cell fragments from mouse retina using a preparation method that yields rod outer segments with large portions of the rod inner segments attached^17^. Therefore, we asked whether Vps34, the major enzyme responsible for PI(3)P synthesis from phosphatidylinositol^18^,^19^, co-fractionates with rhodopsin, the most abundant outer-segment resident marker. Because the outer segment is a modified primary cilium, both its membranes and its cytoplasm have a composition distinct from that of the inner segment. During the preparation, the cells break open, releasing some portion of their cytoplasm, including soluble enzymes like Vps34. However, structural analysis reveals that the breakage point is predominantly in the inner segment^17^, so that rod outer segments largely retain their soluble components. Fractionation by density gradient ultracentrifugation (Fig. 1C) reveals no evidence for co-migration of rhodopsin and Vps34, although there are some off-peak fractions in which both are present. This result indicates that Vps34 is released from the cell fragments, implying that it is localized to the inner segment, rather than the outer segment, and that most of the PI(3)P synthesis is therefore likely to take place in the inner segment. Because many membrane components are synthesized in the inner segment and then vectorally transferred to the outer segment, we localized PI(3)P-containing membranes using PI(3)P binding domains fused to EGFP. This construct was expressed in rods by *in vivo* electroporation in neonatal retinas^20^, and revealed the presence of large brightly stained puncta, exclusively in the inner segment (Fig. 1B). No evidence was found for the presence of PI(3)P in the outer segment.

### Vps34 functional knockout leads to rapid rod cell degeneration

**Figure 2:**
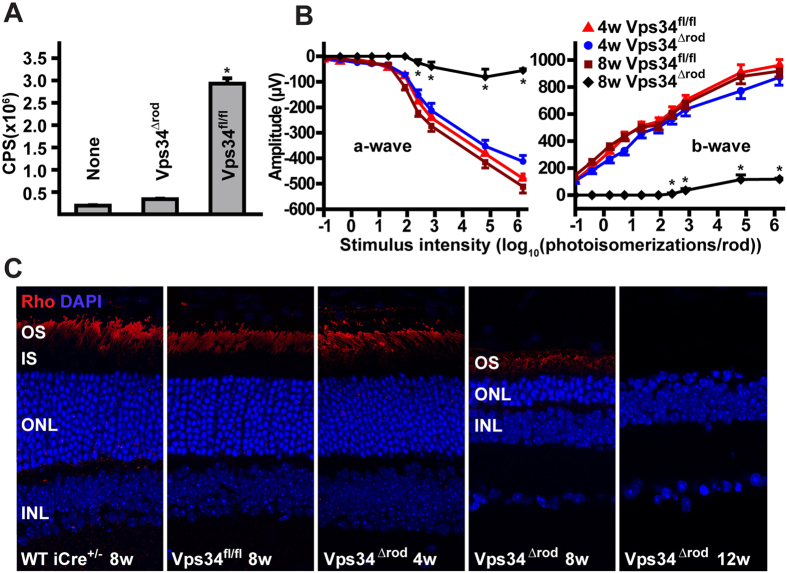
Loss of PI(3)P and rapid retinal degeneration in Vps34^∆rod^ mice. (**A**) PI(3)P ELISA of lipids extracted from rod inner/outer segments of light-exposed retinas. Error bars are means ± SEM, *n* = 3. (**B**) Loss of visual responses revealed by electroretinography (ERG). Error bars are means ± SEM, *n* = 4–6 mice. * indicates a significant difference between 8 week Vps34^fl/fl^ and 8 week Vps34^Δrod^ (p < 0.001). (**C**) Retina sections of control and knockout mice stained with rhodopsin antibodies (Rho) and DAPI reveal loss of more than half of photoreceptors by 8 weeks, and no detectable rods by 12 weeks, in Vps34^∆rod^ animals.

To test the hypothesis that PI(3)P production is essential for photoreceptor function, we generated mice with a rod-specific ablation of functional Vps34, due to an in-frame deletion of the Vps34 P-loop in the ATP binding domain^21^, allowing effects of loss of catalytic activity to be assessed independently of any structural role (Supplementary Fig. S2). We used two different rhodopsin-promoter-driven Cre-expressing transgenes. One of these^22^ expresses Cre in rods in a variable pattern, with expression in at most 50% of cells when tested in the Vps34^flox/flox^ background. The other, iCre-75^23^, expresses Cre in virtually every rod, as verified by immunohistochemistry with Cre antibody (Supplementary Fig. S2). ELISA assays confirmed a reduction in light-driven PI(3)P of approximately 50% in the mice with mosaic Cre expression (Supplementary Fig. S2), and a more than 94% reduction in mice homozygous for the floxed allele in the iCre-75 background (referred to as Vps34^Δrod^; Fig. 2A), consistent with the conclusion that the only major source of PI(3)P in rods is Vps34 phosphorylation of phosphatidylinositol.

At early ages (up to 4 weeks postnatal), the knockout mice had relatively normal retinal structure as observed by light and electron microscopy as well as normal electroretinogram (ERG) responses (Fig. 2B,C). However, they underwent progressive retinal neurodegeneration, with nearly complete loss of rod driven a-wave and b-wave responses in ERGs by eight weeks of age (Fig. 2B). More than half of the photoreceptor nuclei were lost by that age, and almost none could be detected at twelve weeks (Fig. 2C). The mice with mosaic Vps34 knockout had an intermediate phenotype, with some regions of the retina appearing normal, and others undergoing degeneration similar to that observed in the absence of Vps34 activity.

### Autophagolysosome maturation is impaired in the absence of Vps34 function

**Figure 3:**
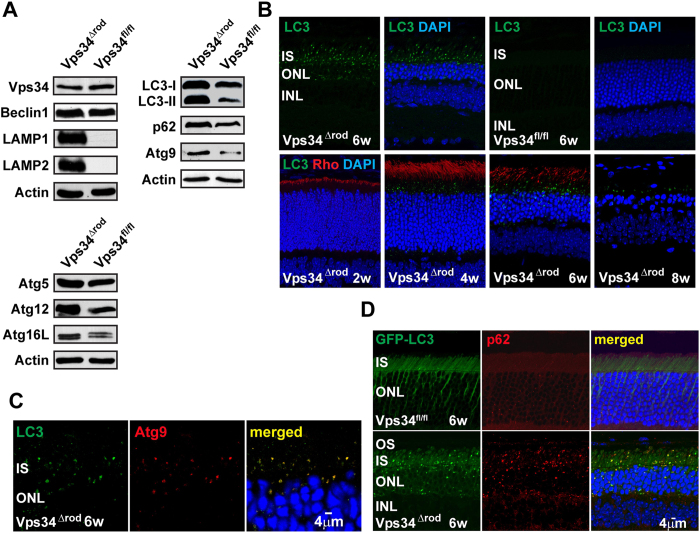
Dysfunction in the autophagy pathway in the absence of Vps34 function. (**A**) Immunoblot reveals increased amounts of autophagy markers LC3/Atg8, Atg9, Atg12, Atg16L and p62, as well as lysosomal markers LAMP1 and LAMP2, in Vps34^∆rod^ retinas. Both LC3-II levels and the ratio of LC3-II/LC3-I are increased. (**B**) LC3-staining puncta accumulated in rods at 4 weeks, prior to detectable changes in retinal structure. (**C**) LC3 co-localized with autophagosomal membrane marker Atg9 in inner segments of Vps34^∆rod^. (**D**) GFP-LC3 and p62 accumulated and co-localized in Vps34^∆rod^-LC3-GFP.

Because of the known role of PI(3)P in autophagy, we used immunoblotting to examine levels of autophagy-related proteins (Fig. 3A). With the exception of Vps34 itself, visible because the knockout allele encodes a nearly full-length protein, and its binding partner Beclin1, all showed substantial increases in 5-week old Vps34^Δrod^ retinas, especially LC3/Atg8, Atg9, Atg12, Atg16L, and p62. There were also large increases in levels of lysosomal markers LAMP1 and LAMP2, which are barely detectable in WT retina. Surprisingly, both the lipidated (LC3-II) and non-lipidated (LC3-I) forms of LC3/Atg8 accumulated to high levels, with the lipidated to non-lipidated ratio increasing in the knockout. Immunofluorescence revealed that the accumulated LC3 is found in large bright puncta, where it co-localizes with Atg9 (Fig. 3C) and p62 (Fig. 3D). These puncta can be observed as early as four weeks postnatal, before any sign of retinal degeneration (Fig. 3B). The accumulation of LC3-containing membranes was confirmed using mice expressing a fusion of EGFP with LC3, which displayed large bright puncta in the absence of active Vps34, but only a diffuse background fluorescence in its presence (Fig. 3D). The GFP-LC3 puncta also co-localized with LC3 antibody staining (Supplementary Fig. S3).

**Figure 4:**
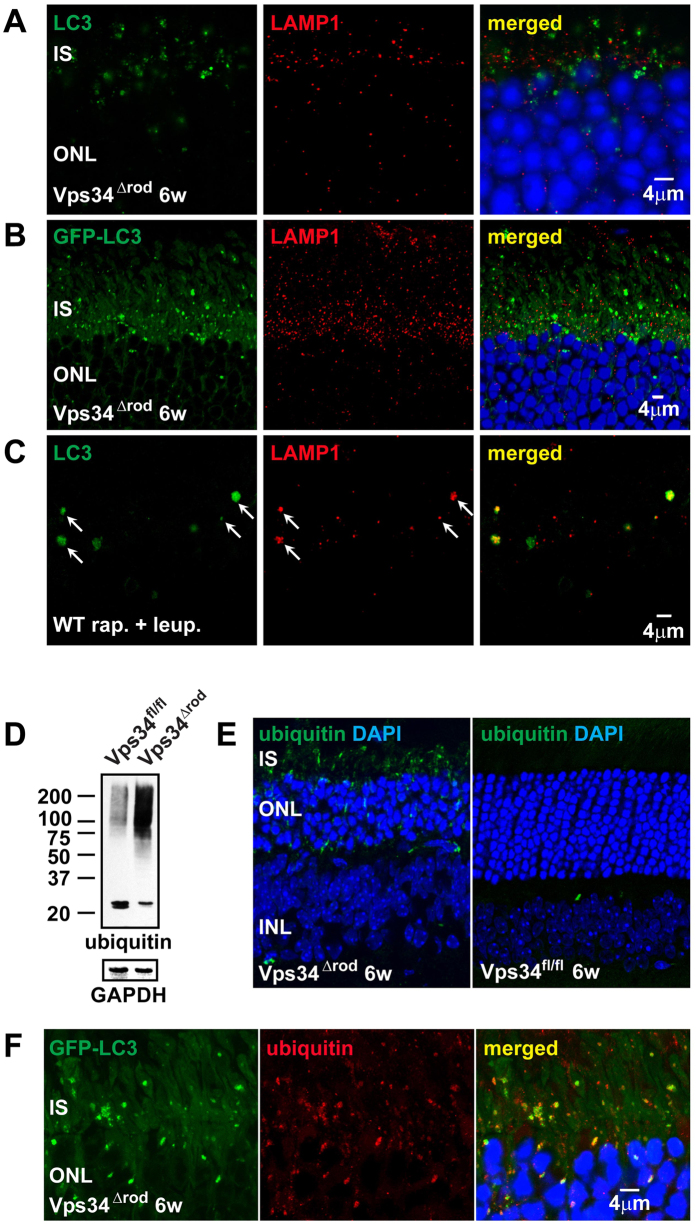
Impairment of autophagolysosome formation in the absence of Vps34 function. (**A**,**B**) The lysosomal marker LAMP1 formed small puncta but did not co-localize either with LC3 or GFP-LC3, indicating defects in autophagolysosome formation. (**C**) LC3 and LAMP1 co-localized in WT C57BL/6 mice that were treated with rapamycin and leupeptin using subretinal injection, indicating autophagolysosome formation in WT but not Vps34^∆rod^ mice. (**D**,**E**) Western blot and immunostaining showing ubiquitinated proteins accumulated in Vps34^∆rod^ retina. (**F**) Co-localization of ubiquitin and LC3 indicates failure of autophagy-mediated degradation of ubiquitinated cargo in Vps34^∆rod^.

These results demonstrate dysfunction in the autophagy pathway in the Vps34^Δrod^. In contrast to previous suggestions that PI(3)P plays a critical role in recruitment of LC3 to early autophagophore membranes^11^,^12^, but consistent with other Vps34 conditional knockouts^24^,^25^,^26^, our results indicate it is dispensable for membrane recruitment and lipidation of LC3. In addition to Atg9, most of the LC3-positive puncta also contain ubiquitinated proteins (Fig. 4E, F), and immunoblots reveal a dramatic increase in high-molecular-weight ubiquitinated species (Fig. 4D). In contrast, the lysosomal marker LAMP1, which also accumulates in the knockout, forms small puncta that do not co-localize with LC3 (Fig. 4A, B). This behavior is distinctly different from that observed in WT mice undergoing autophagy. WT retinas treated with rapamycin to stimulate autophagy, and leupeptin to block lysosomal proteases, also accumulate LC3-positive and LAMP1 positive puncta, but they are much larger, and co-localize (Fig. 4C), implying that the absence of PI(3)P prevents pre-autophagosome structures containing LC3, Atg9, and ubiquitinated cargo from progressing to the stage of lysosomal fusion.

**Figure 5:**
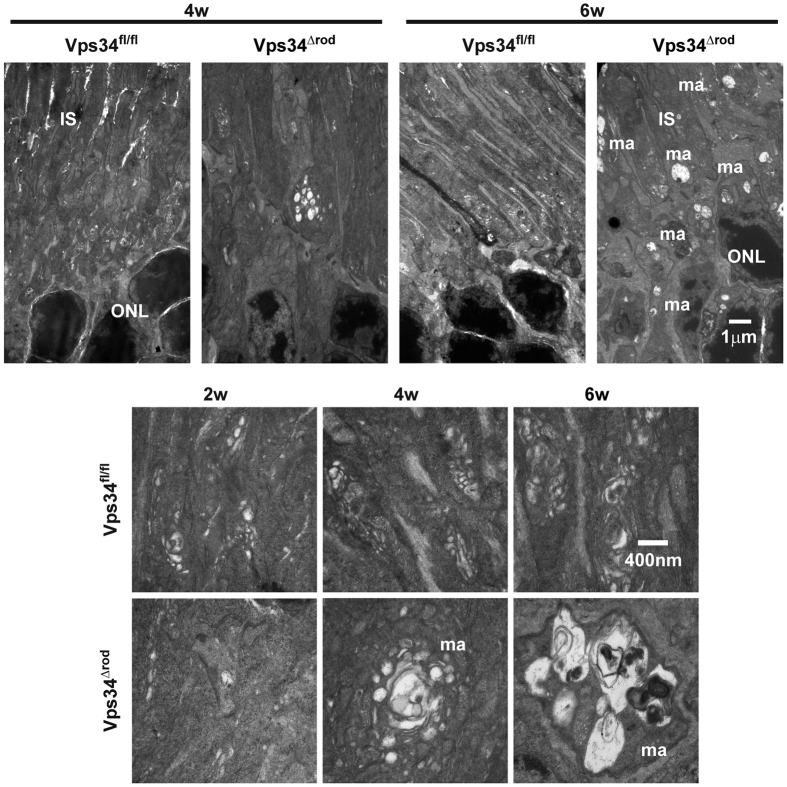
Electron micrographs showed abnormal membrane aggregates formed after loss of Vps34 activity. Retinal samples from 2 to 6 week old mice were imaged at magnifications of 7,000 (top panel) and 30,000 (bottom panel). ma, membrane aggregates; IS, inner segments; ONL, outer nuclear layer.

**Figure 6:**
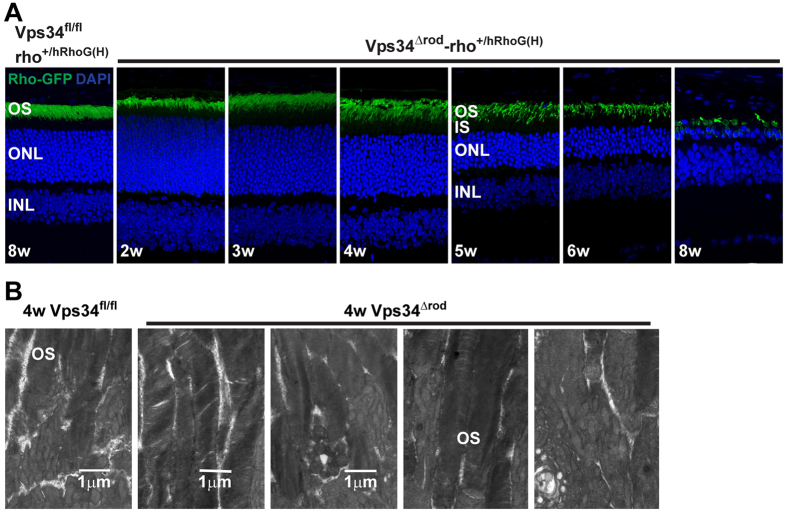
Normal rhodopsin trafficking and disc membranes in the Vps34 knockout. (**A**) hRho-GFP was properly trafficked to the OS and not mis-localized either in IS or ONL in Vps34^∆rod^-rho^+/hRhoG(H)^ mice, indicating little if any effect of the Vps34 KO on rhodopsin trafficking. (**B**) Transmission electron microscopy images at a magnification of 7000× from 4 week old Vps34 floxed retina (*left*) or Vps34^Δrod^ retina (*right*) reveal the formation of normal-appearing disc membranes in outer segments (OS), indicative of normal rhodopsin trafficking and disc assembly in OS.

To determine the structures of membranes formed in the absence of PI(3)P, we examined Vps34^Δrod^ retinas by transmission electron microscopy (TEM). At four weeks of age, the most striking feature was the presence of very large multi-vesicular membrane aggregates (Fig. 5) in Vps34^Δrod^ rods. Although membrane structures with encapsulated vesicles and debris were observed in wild type cells, the very large aggregates present in nearly all fields of Vps34^Δrod^ rods were completely lacking. In neither genotype were there significant numbers of classical double-membrane structures characteristic of autophagosomes, consistent with the observations by immunofluorescence that puncta positive for autophagy markers do not accumulate in WT rods under normal conditions, and that the aberrant structures formed in Vps34^Δrod^ rods, which are positive for these markers, are very large and cannot fuse with lysosomes. At two or four weeks of age, outer segments and disk stacks appeared normal, but at 6 weeks and later the outer segments were shortened, and aberrant membrane structures were observed throughout the cells (Figs 5 and 6B).

### Vps34 knockout rod cells exhibit normal rhodopsin trafficking and disc membrane formation

It has been proposed that PI(3)P is important for trafficking of rhodopsin to the outer segment^3^. To enhance our ability to detect low levels of rhodopsin mis-trafficking, we made use of an extensively characterized mouse line with a knock-in of a gene encoding human rhodopsin fused to EGFP (hRhoG(H))^27^. Rhodopsin localization, and mis-localization of even minor sub-populations of the fusion protein, can be observed with high sensitivity in this strain. Surprisingly, in 4-week-old Vps34^Δrod^-rho^+/hRhoG(H)^ mice, at which time large membrane aggregates have already formed, indicating phenotypic consequences of Vps34 deletion, rhodopsin distribution appears normal, with the rhodopsin fusion protein localized to the outer segment, and virtually none detectable in the inner segment (Fig. 6A). Immunostaining with a monoclonal antibody directed to the carboxyl terminus of rhodopsin (Fig. 3B) led to the same conclusion. Thus, whereas Vps34 and PI(3)P are clearly essential for processes necessary for rod cell function and survival, they do not play an important role in rhodopsin trafficking. Ultrastructural studies support this conclusion (Fig. 6B), since structurally intact outer segments with normal disk structures require proper rhodopsin trafficking^28^. Our TEM results indicate that Vps34 mice have normal disk structure at ages when phenotypic effects of the knockout are readily detectable. The disks appear to have their normal complement of phototransduction proteins, as in the degenerating retina their levels are reduced roughly in proportion to the loss of rhodopsin due to outer segment shortening and cell death (Supplementary Fig. S4).

### Endosome processing is impaired in the absence of Vps34 function

**Figure 7:**
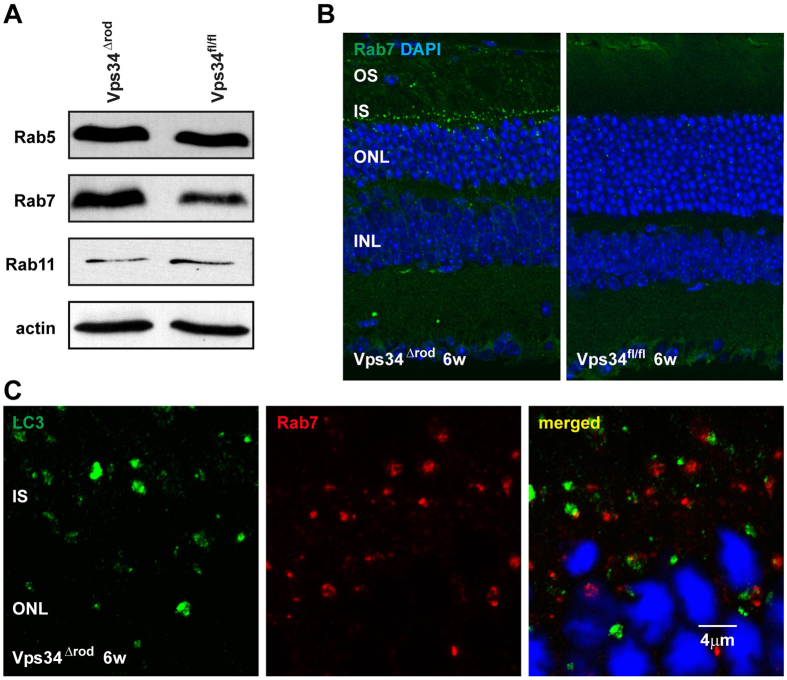
Defect of endosomal pathway in Vps34^∆rod^. (**A**) Immunoblot showed increased levels of late endosome marker Rab7 in Vps34^∆rod^ knockout retinas; same lysate as upper right panel of Fig. 3A. (**B**) Rab7 accumulated in inner segment puncta in Vps34^∆rod^. (**C**) Rab7 puncta do not co-localize with LC3-positive autophagy related membranes.

The onset of obvious structural and functional defects and of cell death is much more rapid in Vps34^Δrod^ retinas than in retinas missing other proteins essential for autophagy^9^,^10^, suggesting that PI(3)P may be important for other processes integral to cell survival. PI(3)P has been implicated in endosome processing and recycling, so we examined markers of early (rab5), recycling (rab11), and late (rab7) endosomes. Levels of rab5 and rab11 were similar to those in floxed rods, but rab7 levels increased substantially in Vps34^Δrod^ rods (Fig. 7A, B). The accumulated rab7 was primarily found in brightly-staining puncta in the inner segment, near the outer nuclear layer, which did not co-localize with the LC3/Atg8-positive membranes (Fig. 7C). They are larger than the LC3 puncta, which in turn are larger than the LAMP1-staining lysosomes (Fig. 4A, B), indicating at least three distinct classes of aberrantly-formed membranes. These results indicate a defect in processing of late endosomes, which is distinct from, and in addition to, the defects in autophagy and suggests that processing of late endosomes is essential for the health and integrity of rod cells.

### Vps34 knockout retinas have an increased number of apoptotic cells

**Figure 8:**
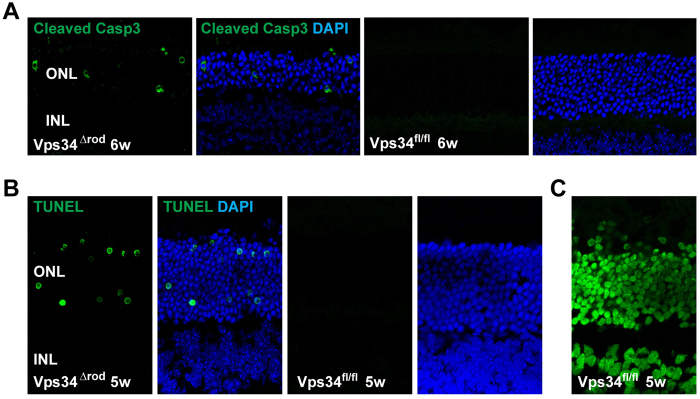
Activation of caspase 3-dependent cell-death pathway in Vps34^Δrod^. (**A**) Immunoactivity toward cleaved caspase 3 increased in the ONL of 6 week Vps34^Δrod^ retina. (**B**) TUNEL assay showed greatly increased numbers of cells with accumulated DNA breaks in rod nuclei in 5 week Vps34^Δrod^ retina. (**C**) WT retina pre-treated with DNase I served as the positive control in the TUNEL assay.

It has been proposed that autophagy and apoptosis are antagonistic and competing processes in many cell types^29^, including photoreceptors^30^. To test for a role of apoptosis in the cell death observed in Vps34^Δrod^ retinas, we used TUNEL staining and immunofluorescence staining with antibodies specific for the cleaved, activated form of caspase 3 (Fig. 8). The results show a clear increase in cells positive for these markers in Vps34^Δrod^ retinas as compared to those containing a normal complement of Vps34. These data support the hypothesis that suppression of Vps34-dependent pathways, including both autophagy and endosome processing, leads to enhanced cell death and retinal neurodegeneration.

### Retinal degeneration in the Vps34 knockout does not require light exposure

Our studies of the effects of rod-specific knockout of Vps34 were motivated by the observation of a light-driven surge in PI(3)P levels in rod cells. However, the neurodegeneration in Vps34^Δrod^ retinas did not require light exposure. Mice maintained in total darkness throughout their lives also suffered from retinal neurodegeneration, and LC3 accumulated in both conditions (Supplementary Fig. S5). To determine whether light exposure might modulate the effects of loss of Vps34, we examined cell loss as a function of position in the retina, and, hence, levels of light exposure, in dark-reared mice, as compared to mice reared in constant light. The results revealed that while degeneration occurred in both conditions, and ultimately involved the entire retina, the rate of progression, in the central retina only, was somewhat greater in the light-reared animals than in the dark-reared animals, whereas in the peripheral retina degeneration occurred with similar kinetics in light or dark (Supplementary Fig. S5). These results reveal that Vps34 is essential for processes required for cell survival in both dark and light conditions, and also for responses to the additional stress induced by light exposure.

### Class I PI-3 kinase knockout in rods does not cause retinal degeneration

**Figure 9:**
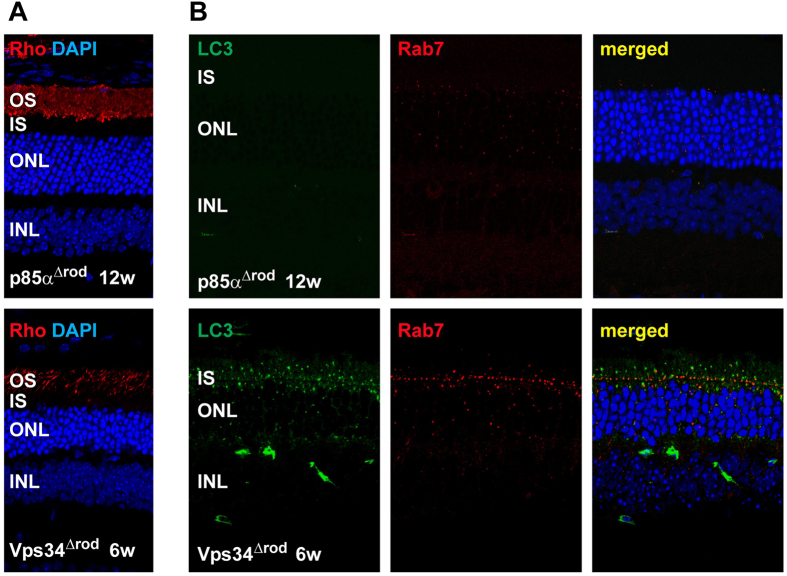
No defect in autophagy or endosomal processing in PI 3-kinase class I knockout mouse retina. (**A**) Normal retina structure was found in 3 month old retina of PI3-kinase class I knockout. (**B**) Neither LC3 nor Rab7 accumulated in puncta in PI 3-kinase class I knockout retina.

Previous studies found no effect of knockout of Class I PI-3 kinase in rods through rod-specific disruption of the gene encoding the regulatory subunit p85α^1^ and we confirmed that those mice had no sign of retinal degeneration. However, our results have revealed that the Cre transgene used in those studies is expressed in only a subset of rods. Therefore, we crossed mice with the floxed allele of p85α with the iCre-75 line, to produce mice with a more complete ablation of p85α. These mice also showed no sign of retinal degeneration (Fig. 9) leading to the conclusion that, in contrast to the type III PI-3 kinase, Vps34, type I PI-3 kinase activity is not important for rod cell health.

## Discussion

Vps34 is thought to be essential for autophagy, which in turn is essential for survival of numerous neurons, including photoreceptors^9^,^10^. In this report, we demonstrate that Vps34 is responsible for a large light-driven increase in PI(3)P levels, and is essential for survival of rods. Ablation of functional Vps34 suppresses PI(3)P synthesis, and leads to aberrant accumulation of large multi-vesicular membrane aggregates in inner segments. By immunofluorescence microscopy, we demonstrated accumulation of inner segment puncta containing autophagy markers but not lysosomal markers, consistent with autophagy intermediates arrested at a stage prior to fusion with lysosomes. The rapid degeneration of Vps34^Δrod^ retinas underscores the importance of PI(3)P. A number of additional novel findings have emerged from this work. In contrast to previous reports, Vps34 and PI(3)P are not essential for rhodopsin trafficking to outer segments and proper formation of outer segments. Furthermore, Vps34 and PI(3)P are not required for formation of autophagosome-related membranes containing the lipidated form of LC3/Atg8, LC3-II, p62, Atg9, and ubiquitinated cargo. Also of significance are the novel findings that defects in endosome processing are lethal to rods, and that both the levels of PI(3)P and cell death resulting from the absence of Vps34 activity are sensitive to light, whose detection with exquisite sensitivity is the major function of rod cells.

## Materials and Methods

### Conditional functional Vps34 and p85α knockout mice

All animal studies were conducted according to NIH guidelines and approved by the Institutional Animal Care and Use Committee at Baylor College of Medicine. WT C57BL/6J mice were purchased from Jackson lab (Bar Harbor, ME). Mice used for tissue samples were euthanized by CO_2_ inhalation prior to dissection.

Vps34 floxed mice^21^ which have lox P sites flanking exons 17 and 18 (the ATP binding domain) were a kind gift from Dr. Fan Wang (Duke University). The C57BL6/129 mixed background Vps34 floxed mice were back-crossed with WT C57BL/6J for at least 6 generations, then bred with transgenic mice containing the opsin promoter controlled iCre-75^23^ in a C57BL/6 background (kindly gifted by Dr. Ching-Kang Jason Chen, Baylor College of Medicine) to generate rod-specific conditional functional Vps34 knockout mice (Vps34^∆rod^). All the experimental Vps34 knockout mice were maintained with heterozygous iCre-75. Vps34 knockout mice that were also heterozygous for the human rhodopsin-GFP allele at the mouse rhodopsin locus^27^ (Vps34^∆rod^-rho^+/hRhoG(H)^) were generated by crossing Vps34^∆rod^ mice with human rhodopsin-GFP knock-in mice containing a loxH site in the 5′ non-coding region to reduce the human rhodopsin-GFP expression by 80%^27^. The Vps34 knockout with heterozygous GFP-LC3 (Vps34^∆rod^-GFP-LC3) was generated by crossing Vps34^∆rod^ mice with C57BL/6 background GFP-LC3 mice (BRC No. 00806: GFP-LC3#53, RIKEN Bio Resource Center, Ibaraki, Japan). In order to make a conditional knockout of class I PI-3 kinase in rods, the BALB/c background p85α floxed mice^1^ (kindly provided by Dr. Raju V. S. Rajala, University of Oklahoma Health Sciences Center, Oklahoma City, OK) were crossed with wild type C57BL/6J for 8–9 generations, then bred with iCre-75 mice to generate rod-specific p85α knockout mice (p85α^∆rod^). Mice with mosaic Vps34 rod knockout (Vsp34^p∆rod^) were generated by crossing Vps34 floxed mice with transgenic mice expressing opsin-promoter-controlled Cre in ~50% of rods^1^ (kindly provided by Dr. Raju V. S. Rajala, University of Oklahoma Health Sciences Center, Oklahoma City, OK).

Clipped tails and/or retinas were used for genotyping as described^21^. PCR primers L1 5′-AACTGGATCTGGGCCTATG-3′ and A2 5′-GAAGCCTGGAACGAGAAGAG-3′ were used for PCR analysis of the deleted Vps34 allele in the retina, which yields a 672 bp PCR product in Vps34 knockout mice (Supplementary Fig. S2). The absence of *rd1* and *rd8* alleles was confirmed by PCR and DNA sequencing as described^31^,^32^.

Unless indicated otherwise, mice were maintained in a 12-12 hour light-dark cycle. The life-time dark- or light-adapted mice were raised in light-proof cages or in 4,500 LUX fluorescent illumination until the mice were dissected.

### Quantification of PI(3)P, PI(3,4)P_2_, and PI(3,4,5)P_3_ with modified ELISA

On the day of the experiment, 20 to 40 4–6 week-old WT C57BL/6 mice were dark-adapted for 12 hours then the mice were exposed to normal fluorescent room light (~540 lux) for the indicated time before the retinas were collected. The rod outer segments (ROS) containing part of the inner segments were collected in 300 μl Ringer’s buffer (10 mM HEPES, 130 mM NaCl, 3.6 mM KCl, 1.2 mM MgCl_2_, 1.2 mM CaCl_2_, 0.02 mM EDTA, pH 7.4) with 8% (v/v) OptiPrep (Sigma). Retinas were vortexed at low speed for 2 min, and then centrifuged at 400 × g for 2 min at room temperature. The supernatants were collected on ice. The process was repeated 5 times followed by layering onto a 10–30% (v/v) OptiPrep gradient and centrifugation for 60 min at 19,210 × g at 4 °C using a TLS-55 rotor (Beckman Coulter). The ROS band was collected, diluted in Ringer’s buffer, and pelleted in a TLS-55 rotor for 30 min at 32,172 × g at 4 °C. The ROS pellet was resuspended in 1 ml Ringer’s buffer and mixed with 1 ml of chloroform:methanol (1:2, v/v), and vortexed for 2 min at room temperature followed by centrifugation for 10 min at 3,661 × g. The lower chloroform phase was collected and transferred to a glass tube. The process was repeated 3 times to remove most of the phospholipids. The samples were then extracted with 1 ml of chloroform:methanol:12N HCl (2:4:0.8, v/v/v), vortexed and centrifuged as above, and the lower chloroform phase was transferred to a glass tube. The chloroform:methanol:HCl extraction was repeated three times, then the chloroform phase was evaporated under an argon stream and stored at −80 °C. Preparations from Vps34^fl/fl^ and Vps34^Δrod^ mice were performed as above, except 10 mice were used for each.

Hepatocyte growth factor-regulated tyrosine kinase substrate (Hrs) PH domain was cloned from purified C57BL/6 mouse kidney mRNA with primers 5′-GCCCTGCTATGAGCAGCTGAACAAGAAGGCA-3′ and 5′-GAAAGTGATGCCATGTTCGCTGCTGAAAGA-3′ with the amfiRivert 1-Step RT-PCR Kit following the manufacturer’s instructions (GenDEPOT, Barker, TX). Mouse tandem PH domain-containing protein-1 (TAPP1) PH domain cDNA^33^ was as gift from Dr. Dario R. Alessi (University of Dundee, Scotland, U.K.) and mouse general receptor of phosphoinositides 1 (GRP1) PH domain cDNA^34^ was kindly gifted by Dr. Mark A. Lemmon (University of Pennsylvania School of Medicine). Two tandem copies of the Hrs or TAPP1 PH domains, or one copy of the GRP1 PH domain, were cloned with a C-terminal 1D4 epitope tag (TETSQVAPA) by PCR, then sub-cloned into the pGEX vector (GE Life Sciences), thereby adding an N-terminal GST tag, yielding the final GST-2xHrs-1D4, GST-2xTAPP1-1D4 and GST-GRP1-1D4 constructs. All recombinant GST-PH domain-1D4 fusion proteins were expressed in BL21(DE3)pLysS *E. coli* (Novagen) at room temperature for 4 hours following induction with 1 mM isopropyl β-D-1-thiogalactopyranoside (IPTG). Protein purification was performed with Glutathione Sepharose 4 Fast Flow (GE Healthcare) following the manufacturer’s protocol, and purity was assessed by SDS-PAGE. Purified proteins were dialyzed against 20 mM Tris pH 7.5, 100 mM NaCl, and phenylmethanesulfonyl fluoride (PMSF) then concentrated, supplemented with 50% glycerol and PMSF, and stored at −20 °C.

The ELISA assay^35^ was modified for determination of phosphoinositides in mouse rod inner/outer segments. Dried phosphoinositide extract was dissolved in 200 μl of chloroform:methanol:MQ water (1:2:0.8, v/v/v), and then diluted with methanol to a final chloroform:methanol ratio of 1:9 (v/v). Total phospholipids were determined using an inorganic phosphorous assay^36^ and used to normalize the phosphoinositide extract. For the PI(3)P, PI(3,4)P_2_ and PIP_3_ standard (Echelon Biosciences, Salt Lake City, UT), 40 μl of sample containing 9.7 fmol to 10 pmol phosphoinositide standard and 128 pmol phospholipid mixture (PC:PE:PS = 45:35:15 molar ratio) was added into each well of an Immulon 2HB 96-well plate (Thermo). For experimental samples, about 4,200 pmol of total phospholipids were added to each well. All samples and standards were performed in triplicate. The lipids were air-dried and then dried under vacuum overnight. The plate was blocked with 5% BSA (Sigma) in PBS for 4 hours at room temperature, and then incubated with purified GST-PH domain-1D4 at 1 μg/ml with 3% BSA in PBS at 4 °C overnight. The plate was washed with PBS 10 times followed by incubation with monoclonal 1D4 antibody^37^ at 1 μg/ml in PBS with 3% BSA at room temperature for 2–3 hours. After incubation with 1D4 antibody, the plate was washed with PBS 10 times, and then incubated with 0.24 μg/ml goat anti-mouse-HRP (Thermo) in PBS with 3% BSA at room temperature for 1 hour. After washing 10 times in PBS, the plate was incubated with 100 μl/well SuperSignal ELISA Femto Maximum Sensitivity Substrate (Thermo) at room temperature for 1 min, and photons were detected using a Victor3 Multilabel Plate Counter (Perkin Elmer). The limit of detection was about 0.1-0.2 pmol.

## Additional Information

**How to cite this article**: He, F. *et al.* Phosphatidylinositol-3-phosphate is light-regulated and essential for survival in retinal rods. *Sci. Rep.* **6**, 26978; doi: 10.1038/srep26978 (2016).

## Supplementary Material

Supplementary Information
