# Supplementary material for: Phosphatidylinositol-3-phosphate is light-regulated and essential for survival in retinal rods
Source: Sci Rep. 2016 Jun 1;6:26978. doi: 10.1038/srep26978 (PMC4887901; doi:10.1038/srep26978)
Supplement: Supplementary Information [file srep26978-s1.pdf]

Phosphatidylinositol-3-phosphate is light-regulated and essential for survival in retinal rods

Feng He[1], Melina A. Agosto[1], Ivan A. Anastassov[1], Dennis Y. Tse[2], Samuel M. Wu[2], Theodore G. Wensel[1,2,*]

1. Verna and Marrs McLean Department of Biochemistry and Molecular Biology and
2. Department of Ophthalmology, Baylor College of Medicine, One Baylor Plaza, Houston, TX, 77030, USA

Supplementary Information

Supplementary Methods

Sub-retinal injection and electroporation

The 2xHrs cDNA was sub-cloned into the pCAG-EGFP vector, which was provided by Dr. Connie Cepko (Addgene plasmid # 11150) and uses the chicken beta-actin promoter with CMV enhancer to drive expression[1], to generate a GFP-2xHrs fusion. Plasmids were introduced by sub-retinal injection and electroporation of P0 mice as described by Cepko et al.[1]. Briefly, newborn mice were anesthetized by chilling on ice, the future edge of the eyelid was cut with the tip of a 30-gauge needle, and a pilot hole through the sclera near the cornea was made using a 30-gauge needle. Approximately 0.45 μl of pCAG-GFP-2xHrs plasmid DNA (2-4 mg/ml) in PBS with 0.1% fast green dye were injected into the sub-retinal space using a Nanofil 10 μl syringe with a 33-gauge blunt-ended needle controlled by a Micro4 MicroSyringe pump controller (World Precision Instruments, Sarasota, FL) under a Nikon SMZ800 dissecting microscope. After injection, tweezer-type electrodes were placed on both eyes and 5 square 80 V pulses of 50 ms duration were applied at 1 s intervals using a homemade pulse generator or an ECM 830 electroporation system (BTX Harvard Apparatus, Holliston, MA). The injected eyes were dissected 3 weeks later and fixed with 4% paraformaldehyde in PBS for 45 min at room temperature followed by embedding in optimum cutting temperature (OCT) compound and cryosectioning.

For subretinal injection of rapamycin, 6 week-old C57BL/6 or CD-1 (Charles River) mice were anaesthetized by intraperitoneal injection of ketamine (100 mg/kg) and xylazine (16.5 mg/kg), and approximately 0.45 μl of DMSO containing 100 μM rapamycin (Selleckchem, Houston, TX), 300 μM leupeptin (Calbiochem), and 0.1% fast green was injected into the sub-retinal layer as above. The injected mice were dissected 10-12 h after injection. The mouse

eyes were rinsed with PBS and immediately embedded into OCT without fixation. Cryosections were then immunostained for LAMP1 and LC3 as described below.

Immunoblotting

Isolated mouse retinas suspended in 2x SDS-PAGE sample buffer, 10 mM EDTA, 4 mM DTT and 2x cOmplete protease inhibitor cocktail (Roche, Indianapolis, IN) were sonicated on ice for 2 minutes. The insoluble material was removed by centrifugation at 17,000 x g for 15 minutes at 4°C. The samples were separated by SDS-PAGE and transferred to nitrocellulose membrane. Membranes were blocked with 5% non-fat milk in TBST (20 mM Tris-HCl, pH 7.6, 150 mM NaCl and 0.1% (v/v) Tween 20) for 1 hour at room temperature, then incubated at 4°C overnight in the blocking solution containing primary antibody. The following antibodies were used for immunoblotting and immunostaining: purified monoclonal 1D4[2] was prepared in-house from hybridoma culture medium; rabbit anti-transducin alpha (K-20), goat anti-PDEβ, rabbit anti-PIK3C3 (Vps34) (H-300), mouse anti-LAMP1 (LY1C6), mouse anti-LAMP2 (H4B4), and mouse anti-actin were from Santa Cruz Biotechnology; RGS9-1 (R4433)[3] and Gβ5 (G4718)[4] antibodies were produced at Bethyl Laboratories; rabbit polyclonal anti-atg3, and Beclin-1 (D40C5), atg7 (D12B11), atg5 (D5F5U), atg12 (D88H11), atg16L1 (D6D5), p62 (D5E2), LC3A/B (D3U4C), Rab5 (C8B1), Rab7 (D95F2), Rab11 (D4F5), and cleaved capspase 3 (5A1E), which are all rabbit monoclonal antibodies, were from Cell Signaling; rabbit anti-Cre recombinase and mouse monoclonal anti-ubiquitin were from Millipore; guinea pig anti-p62 for immunostaining was from American Research Products; mouse anti-LC3 was from MBL; and rabbit anti-Atg9 was from Abcam. The membranes were washed three times with TBST and incubated with goat anti-mouse/rabbit IRDye (LI-COR Biotechnology, Lincoln, NE) or goat anti-mouse/rabbit-HRP (Jackson ImmunoResearch Laboratories) secondary antibody diluted 1:10,000 in blocking buffer, washed with TBST buffer, and then imaged and analyzed with an Odyssey infrared

imaging system (LI-COR Biotechnology) or incubated with enhanced chemiluminescence reagent (Pierce) and exposed to film. All western blots shown are representative examples of three or more experiments.

Immunofluorescence staining and microscopy

Mouse eyes were excised and rinsed with BPS. The eyes were fixed with 4% paraformaldehyde in PBS for 45 min at room temperature then cryoprotected with 26% sucrose in PBS at 4ºC overnight, and embedded in OCT. For immunostaining with LAMP1 antibody, the mouse eyes were embedded in OCT directly without fixation. 20 μm sections were cut using a cryo-microtome (Fisher). The unfixed sections were post-fixed with 1% paraformaldehyde in PBS for 2-3 min at room temperature. All the sections were washed with PBS 5-6 times before blocking with 20% donkey serum, 5% BSA, 0.5% fish gelatin, and 0.2% triton-X100 in PBS at room temperature for 1 hour. For anti-Cre recombinase immunostaining, the antigen retrieval procedure was performed as follows: after the PBS washes, the sections were immersed in citrate buffer, pH 6.0 (Diagnostic BioSystem) and incubated at 100ºC for 30 min. The sections were cooled to room temperature, rinsed with deionized water, and washed with PBS before blocking. The sections were incubated with primary antibodies that were diluted 1:50-1:100 with blocking solution at 4ºC overnight. After washing with PBS 6-8 times, the sections were incubated with secondary antibody conjugated with Alexa Fluor 488 or 555 (Life Technologies) at 1 μg/ml and 300 nM DAPI in blocking solution for 1 hour at room temperature. The sections were then washed with PBS 8 times and mounted with coverslips and Vectashield anti-fade reagent (Vector Laboratories).

Samples were imaged with a Leica TCS SP5 laser scanning confocal microscope system at room temperature using a 63x oil immersion objective (Leica, HC PL APO CS2 63.0x, numerical aperture 1.40) with the 405 nm diode laser, 488 nm argon laser, and 543 nm HeNe

laser. Z-stacks were acquired at 1024 x 1024 pixels in 0.1 μm steps, and converted to maximum z-projections using LAS AF software (Leica). Images were converted to tiff format using ImageJ, and contrast and brightness were adjusted with Photoshop (Adobe). All immunostaining images shown are representative examples of three or more experiments.

Electroretinogram (ERG) recordings

ERGs were recorded bilaterally from Vps34 KO mice and their floxed littermates at ages 4 and 8 weeks. Mice were allowed to dark-adapt overnight. Prior to testing, mice were anaesthetized under dim red light with intraperitoneal injection of a solution containing ketamine (95 mg/kg), xylazine (5 mg/kg), and acepromazine. One drop of 1% tropicamide, 2.5% phenylephrine, and 0.5% proparacaine hydrochloride was instilled on each eye for mydriasis of the pupil and local anesthesia of the cornea. Mice were placed inside a Ganzfeld dome coated with highly reflective white paint (Munsell Paint, New Windsor, NY), where a heating pad maintained their body temperature at 39°C. A blunt platinum rod electrode was placed in contact with the center of each cornea as the active electrode. A drop of 2.5% methylcellulose gel (Goniosoft, Ocusoft Inc, TX) was applied to the eye to improve conductivity and to maintain hydration of the cornea. Ground and reference platinum electrodes were gently inserted under the skin at the tail and the forehead, respectively. The mice were then allowed to remain in complete darkness for 5 minutes before recording.

For scotopic a-wave and b-wave measurements, 0.5 ms square flashes of 503 nm peak wavelength were produced by cyan light emitting diodes. All flashes were calibrated with a photometer (ILT1700, International Light, Peabody, MA) and converted to units of photoisomerizations/rod[5,6], where 1 scot cd m^2 = 581 photoisomerizations/rod/s. Signal averaging was employed during recording in which 40 responses were averaged with a delay of 2 s between each at the lowest intensity. As the intensity of the flash increased, fewer

responses were averaged with a longer delay between flashes. A pair of 1500 W xenon lamps (Novatron, Dallas, TX) attenuated with apertures and diffusers were used to produce two saturating light stimuli at the end of the scotopic protocol.

Electrical signals were amplified with a strain gage amplifier (P122, Grass Instruments, West Warwick, RI) and band-pass filtered from 0.1 to 1,000 Hz. Signal was digitized at a 10 kHz sampling rate with a National Instruments data acquisition unit (USB-6216, National Instruments, Austin, TX), and analyzed with custom Matlab code (MathWorks, Natick, MA). The b-wave was digitally filtered using the filtfilt function in Matlab (low-pass filter; Fc = 60 Hz) to remove oscillatory potentials. The a-wave was measured from baseline to the trough of the initial negative deflection (unfiltered) and the b-wave was measured from the a-wave trough to the peak of the subsequent positive deflection (filtered).

Transmission electron microscopy

Mouse eyes were enucleated and the retina was left attached to the choroid to preserve photoreceptor outer segment morphology. The resulting eye cup was fixed with 3% paraformaldehyde and 3% glutaraldehyde solution in 0.1M cacodylate buffer, pH 7.4 (CB) overnight to several days at 4ºC. Eyecups were washed and post-fixed with 1% OsO_4 in 0.1M CB for 1 hr, followed by graded dehydration in fresh ethanol (30%, 50%, 70%, 85%, 90%, and 100%). Subsequently, the tissue was rotated in several changes of acetone and gradually infiltrated with 2:1, 1:1 and 1:2 acetone to resin (Embed-812, Electron Microscopy Sciences, Hatfield, PA) mixture. Infiltration with pure resin for 48 h followed. Tissue was oriented in molds and resin blocks were polymerized at 60ºC for 24-48 h. Ultra-thin sections (80-90nm) were cut on a Leica Ultracut UCT microtome, collected on 50 or 75 mesh grids and stained with 2% uranyl acetate and Reynold's lead citrate. Images were taken on a Zeiss transmission electron

microscope equipped with a CCD camera (Advanced Microscopy Techniques Corp., Woburn, MA).

Statistical analysis

Data were analyzed using unpaired two-tailed t-tests with Prism v.3 software (GraphPad) and Origin v.7.5 (OriginLab).

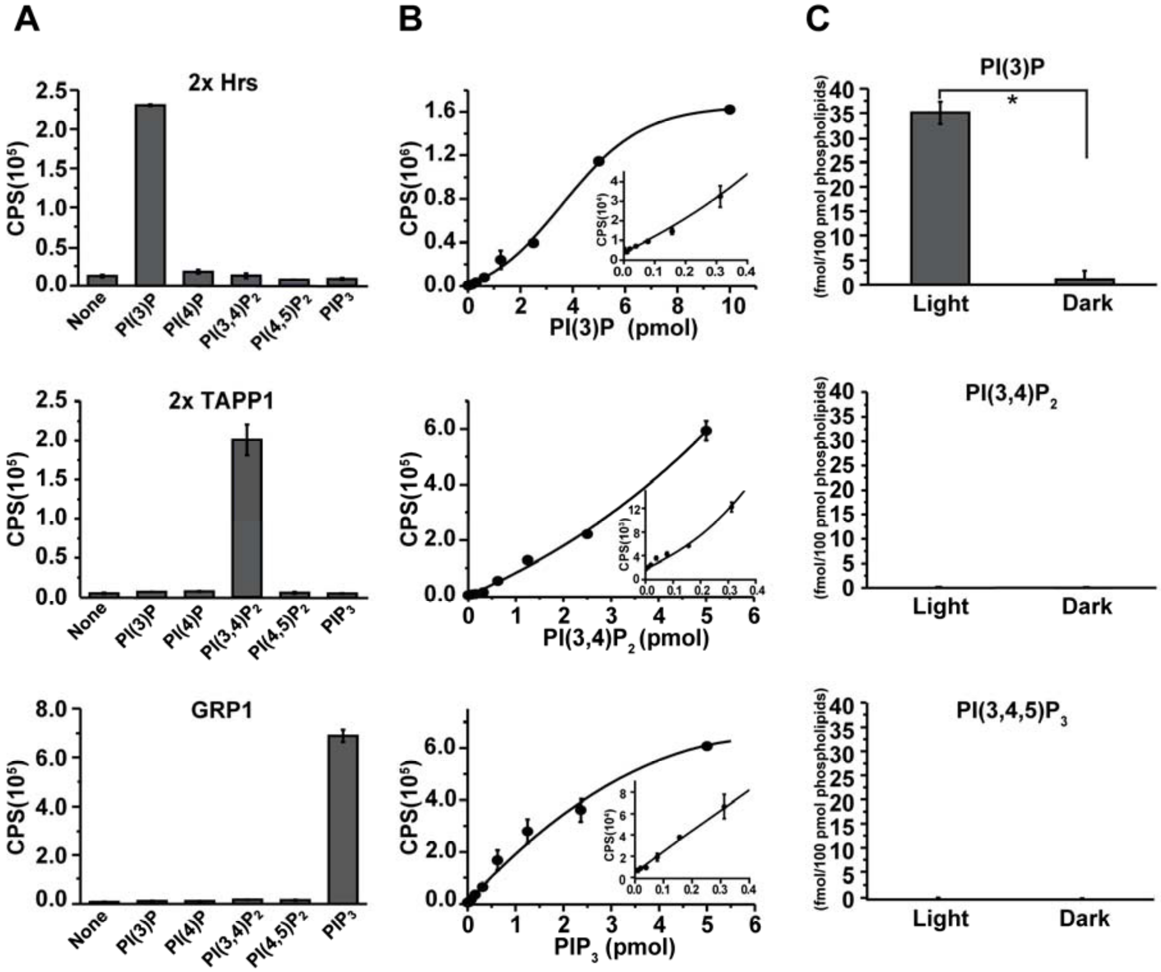

Supplementary Figure S1. **Specificity and sensitivity of the ELISA assay for PI(3)P, PI(3,4)P_2, and PI(3,4,5)P_3.** (**A**) The specificity of purified recombinant PH domains for their target phosphoinositides was verified using a panel of synthetic phosphoinositides. Each well contained 2.5 pmol of the indicated phosphoinositide and 100 pmol of PC:PE:PS mixture (50:35:15). Wells were incubated with purified 1D4-tagged PH domains, followed by detection using the ELISA assay as described in Materials and methods. (**B**) Standard curves were generated for each phosphoinositide. Inserts show a magnified view of the standard curves below 0.4 pmol. Data in (A) and (B) represent means ± SEM of triplicates from a representative experiment. (**C**) 4,200 pmol of purified phospholipids from light- or dark-adapted mouse rod inner/outer segments were subjected to the ELISA assay, and phosphoinositides were quantified using the standard curves in (**B**). PI(3,4)P_2 and PI(3,4,5)P_3 were not detectable in our experimental conditions. Data are mean ± SEM of three experiments, each performed in triplicate. *, $p<0.001$. For details, see *Materials and methods*.

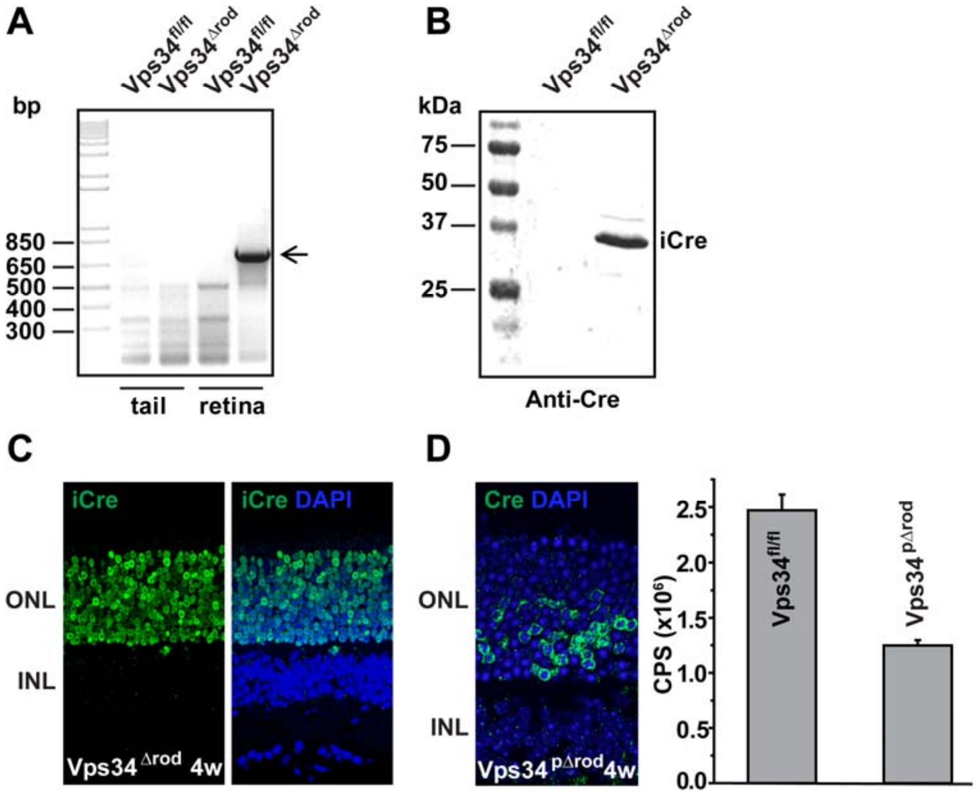

Supplementary Figure S2. **Analysis of Cre recombinase expression.** (**A**) PCR analysis of the Vps34$^{\Delta rod}$ allele. PCR analysis with primers L1 and A2 (see Materials and methods) indicates the presence of the deleted allele (672 bp product) only in the Vps34$^{\Delta rod}$ retina. (**B**) Western blot showing Cre expression in Vps34$^{\Delta rod}$ retinas. (**C**) Cre immunostaining of Vps34$^{\Delta rod}$ retinas shows iCre75 recombinase is present in virtually every rod and expression is restricted to the ONL. (**D**) Cre immunostaining of retina from a mouse homozygous for the floxed allele of Vps34 and heterozygous for the rhodopsin-Cre allele with mosaic expression (Vsp34$^{p\Delta rod}$). ELISA analysis of inner/outer segments from light-exposed WT and Vsp34$^{p\Delta rod}$ retinas showed a ~50% reduction of PI(3)P in the mosaic strain.

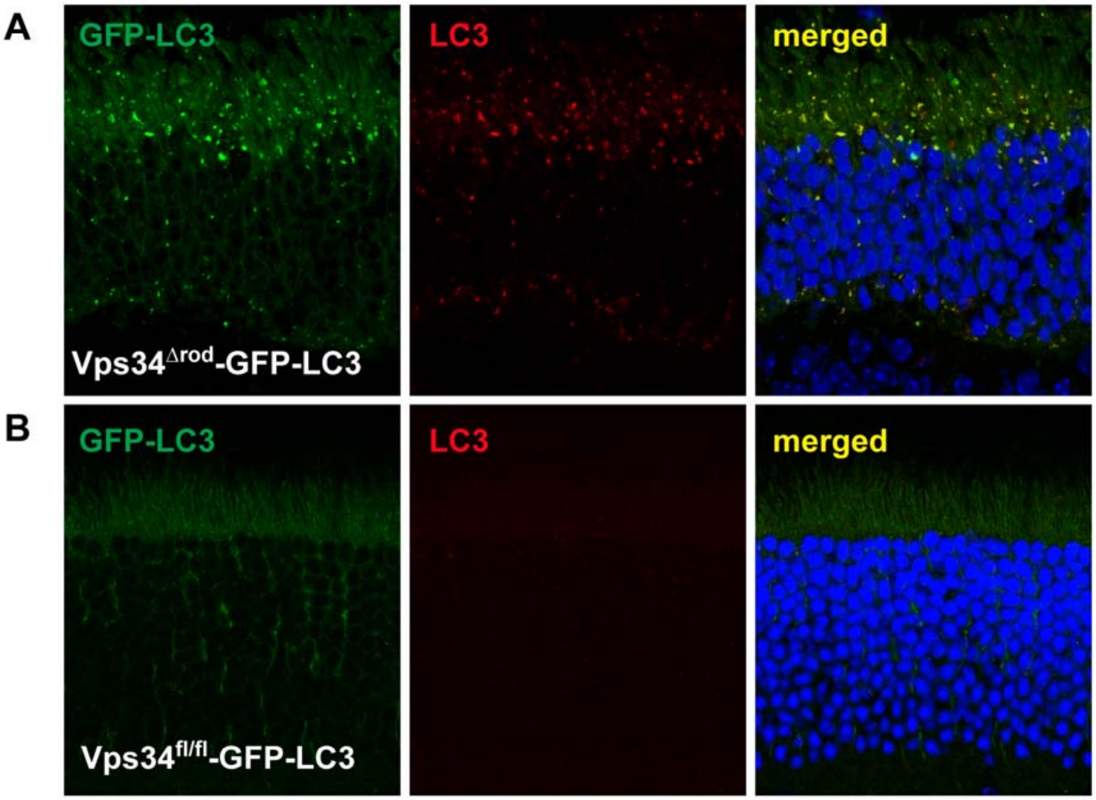

Supplementary Figure S3. **Immunostaining of GFP-LC3 with LC3 antibody.** (**A**) GFP-LC puncta accumulated in 6 week Vps34$^{\Delta rod}$-GFP-LC3 mouse retina and co-localized with LC3 antibody immunostaining. (**B**) GFP-LC3 puncta did not accumulate in Vps34$^{fl/fl}$-GFP-LC3 retina.

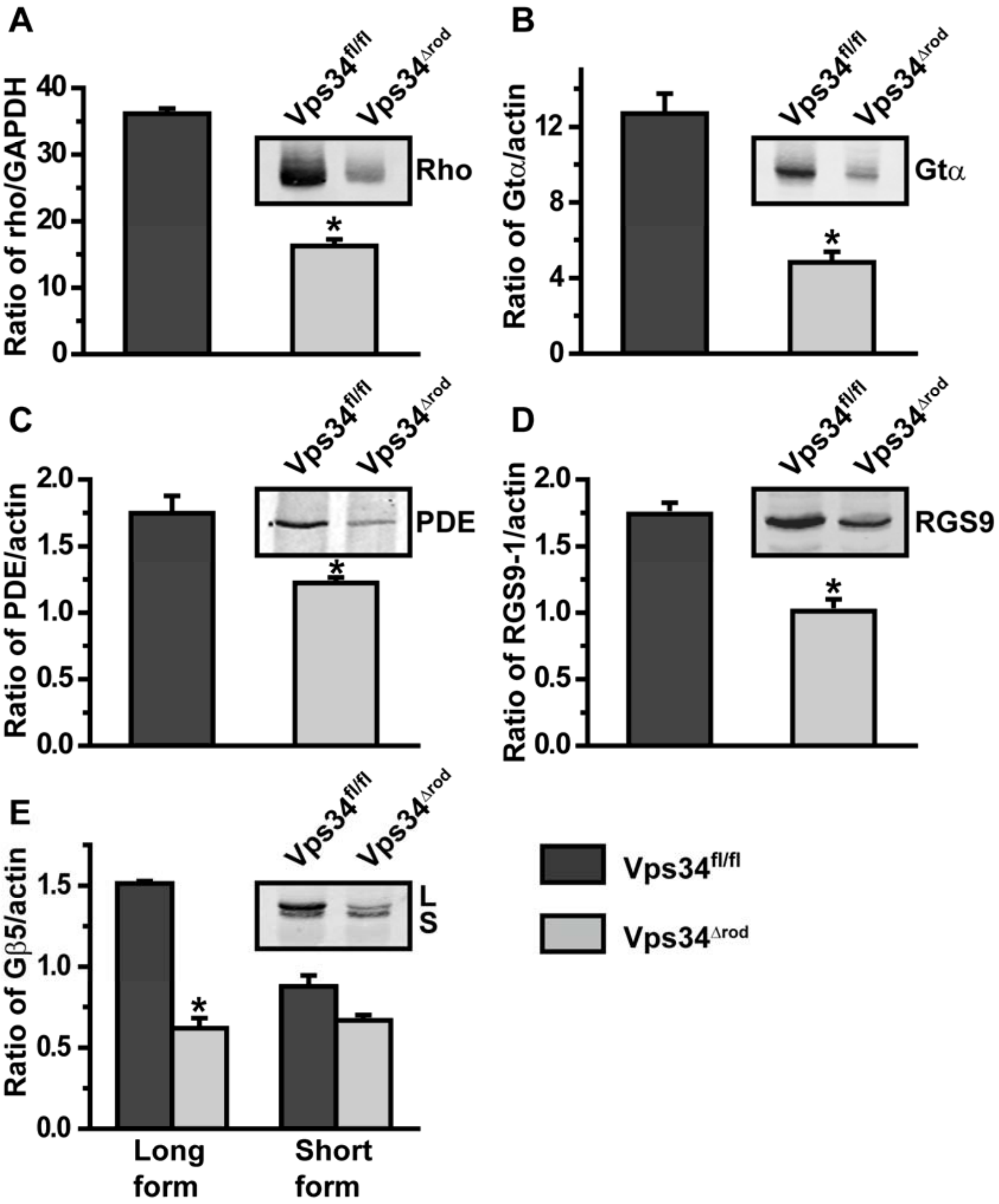

Supplementary Figure S4. **Phototransduction proteins in Vps34$^{\Delta rod}$ retina.** (**A**-**E**) The levels of rhodopsin, transducin, PDEβ, RGS9, and Gβ5 were reduced roughly in proportion to the loss of rods due to rod degeneration. L: long form Gβ5, S: short form Gβ5 in (**E**). Data are means ± SEM of three independent experiments, each performed with retinas from three mice, which were pooled and blotted in triplicate lanes. *, $p<0.05$, compared with control Vps34$^{fl/fl}$ samples.

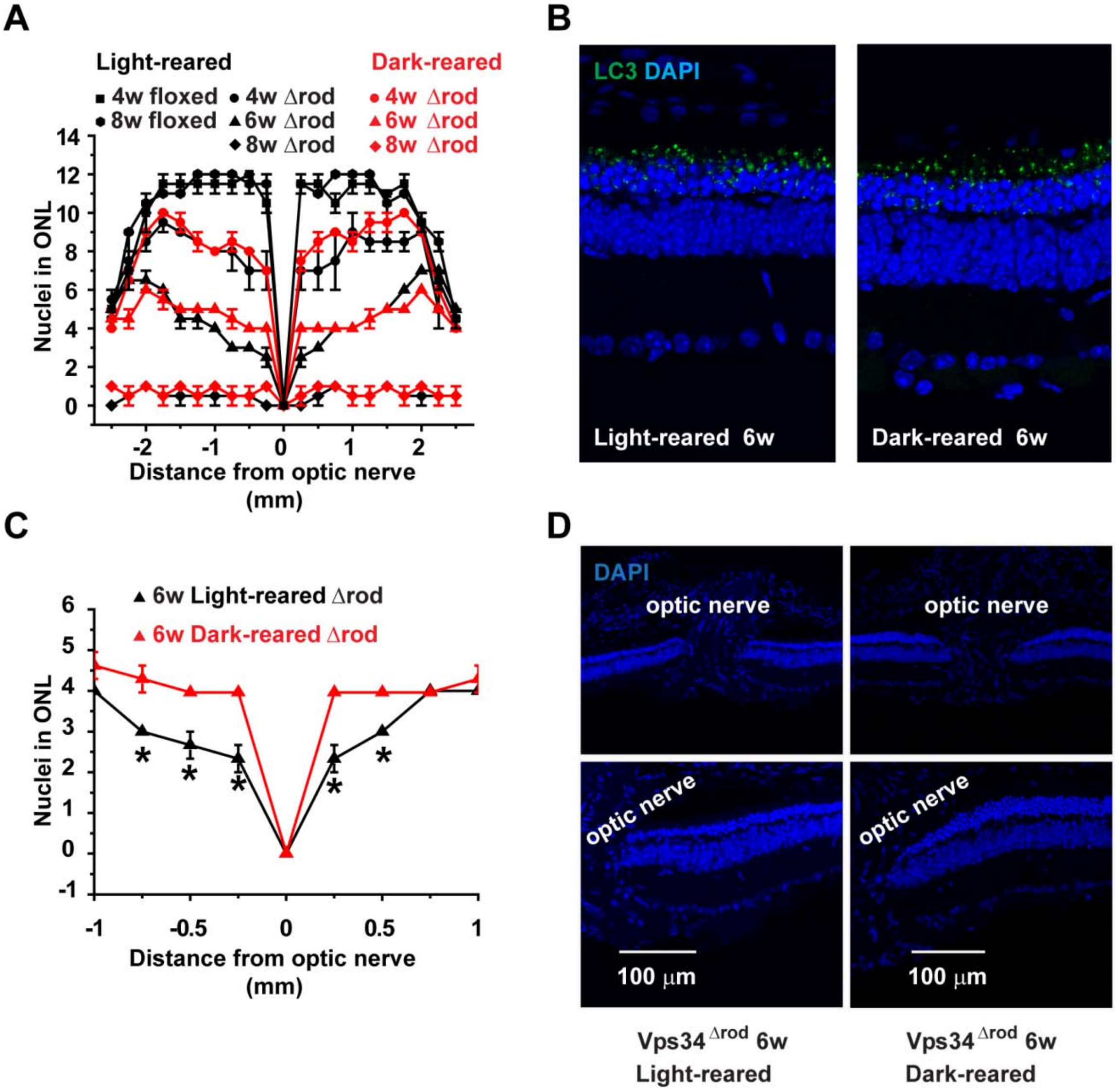

Supplementary Figure S5. **Effects of bright light-rearing versus dark-rearing on Vps34$^{\Delta rod}$ retina.** (**A**) "Spidergram" plots of ONL thickness vs. distance from optic nerve in 4, 6, or 8 week old light- or dark-reared Vps34 floxed and Vps34$^{\Delta rod}$ mice. Dark-rearing did not prevent retinal degeneration in Vps34$^{\Delta rod}$. Data are mean ± SEM, n = 4 retina for each age group of light-reared Vps34$^{fl/fl}$, light-reared Vps34$^{\Delta rod}$ and dark-reared Vps34$^{\Delta rod}$. (**B**) Immunostaining of LC3 revealed similar levels of accumulated puncta in 6 week old light-and dark-reared Vps34$^{\Delta rod}$ mice. (**C**,**D**) The degeneration of the central retina at 6 weeks postnatal was more advanced in bright light-reared Vps34$^{\Delta rod}$ mice than in 6 week old dark-reared Vps34$^{\Delta rod}$ mice (*, $p<0.05$).

Supplementary References

1. Matsuda, T. & Cepko, C. L. Electroporation and RNA interference in the rodent retina in vivo and in vitro. *Proc. Natl. Acad. Sci. U.S.A.* **101,** 16-22 (2004).
2. Molday, R. S. & MacKenzie, D. Monoclonal antibodies to rhodopsin: characterization, cross-reactivity, and application as structural probes. *Biochemistry* **22,** 653-660 (1983).
3. Cowan, C. W., He, W. & Wensel, T. G. RGS proteins: lessons from the RGS9 subfamily. *Prog. Nucleic. Acid. Res. Mol. Biol.* **65,** 341-359 (2001).
4. Mancuso, J. J., Qian, Y., Long, C., Wu, G. Y. & Wensel, T. G. Distribution of RGS9-2 in neurons of the mouse striatum. *J. Neurochem.* **112,** 651-661 (2010).
5. Abd-El-Barr, M. M. *et al.* Genetic dissection of rod and cone pathways in the dark-adapted mouse retina. *J. Neurophysiol.* **102,** 1945-1955 (2009).
6. Frankfort, B. J. *et al.* Elevated intraocular pressure causes inner retinal dysfunction before cell loss in a mouse model of experimental glaucoma. *Invest. Ophthalmol. Vis. Sci.* **54,** 762-770 (2013).
